# The porcine circovirus type 1 capsid gene promoter improves antigen expression and immunogenicity in a HIV-1 plasmid vaccine

**DOI:** 10.1186/1743-422X-8-51

**Published:** 2011-02-07

**Authors:** Fiona L Tanzer, Enid G Shephard, Kenneth E Palmer, Marieta Burger, Anna-Lise Williamson, Edward P Rybicki

**Affiliations:** 1 Department of Molecular and Cell Biology, Faculty of Science, University of Cape Town, Rondebosch, Cape Town, 7701 South Africa; 2 Institute of Infectious Disease and Molecular Medicine, Faculty of Health Sciences, University of Cape Town, Observatory, Cape Town, 7925 South Africa; 3 Department of Medicine, Faculty of Health Sciences, University of Cape Town, Observatory, Cape Town, 7925 South Africa; 4 Department of Pharmacology and Toxicology & James Graham Brown Cancer Center, University of Louisville School of Medicine, 505 South Hancock Street, Louisville KY 40202, USA; 5 National Health Laboratory Service, Groote Schuur Hospital, Observatory, Cape Town, 7925 South Africa

## Abstract

**Background:**

One of the promising avenues for development of vaccines against Human immunodeficiency virus type 1 (HIV-1) and other human pathogens is the use of plasmid-based DNA vaccines. However, relatively large doses of plasmid must be injected for a relatively weak response. We investigated whether genome elements from Porcine circovirus type 1 (PCV-1), an apathogenic small ssDNA-containing virus, had useful expression-enhancing properties that could allow dose-sparing in a plasmid vaccine.

**Results:**

The linearised PCV-1 genome inserted 5' of the CMV promoter in the well-characterised HIV-1 plasmid vaccine pTHgrttnC increased expression of the polyantigen up to 2-fold, and elicited 3-fold higher CTL responses in mice at 10-fold lower doses than unmodified pTHgrttnC. The PCV-1 capsid gene promoter (Pcap) alone was equally effective. Enhancing activity was traced to a putative composite host transcription factor binding site and a "Conserved Late Element" transcription-enhancing sequence previously unidentified in circoviruses.

**Conclusions:**

We identified a novel PCV-1 genome-derived enhancer sequence that significantly increased antigen expression from plasmids in *in vitro *assays, and improved immunogenicity in mice of the HIV-1 subtype C vaccine plasmid, pTHgrttnC. This should allow significant dose sparing of, or increased responses to, this and other plasmid-based vaccines. We also report investigations of the potential of other circovirus-derived sequences to be similarly used.

## Background

Plasmid vaccines are increasingly accepted as being useful for priming cytolytic T lymphocyte (CTL) responses against pathogens in heterologous prime-boost vaccine regimens [1-3]. However, while plasmid vaccines elicit strong cellular immune responses in small mammals, they have elicited less potent responses in clinical trials, and large or repeated doses appear to be necessary in order to prime strong CTL responses in humans [4].

The antigen-encoding transgene in a plasmid vaccine vector for mammalian use is expressed intracellularly in vaccinated hosts, under the control of mammalian cell-compatible transcription and mRNA processing signals, including promoters, Kozak sequences, introns, and polyadenylation sequences [5]. A number of vaccine design modifications are being investigated with a view to improving plasmid vaccine immunogenicity and thereby reducing the dose necessary for a strong CTL response [2,5,6]. One approach is to improve expression of the plasmid encoded antigen so that smaller doses will still elicit effective immune responses.

**Figure 1:**
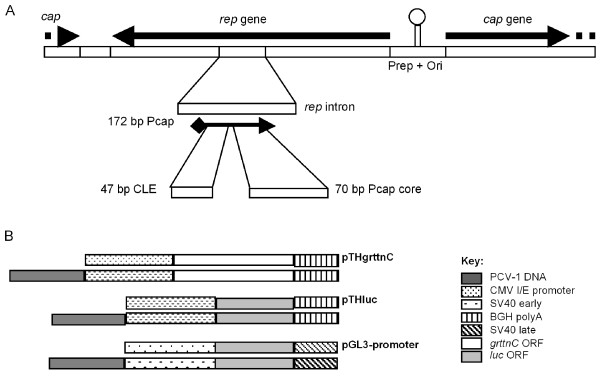
**PCV genome arrangement and cloning scheme**. **A**. Diagram of the linearised PCV-1 genome, depicted in the orientation cloned into pTHCapgrttnC. The *rep *intron is shown enlarged, and the capsid gene promoter (Pcap) is indicated. The core and Conserved Late Element (CLE) components of Pcap are shown enlarged. Abbreviations: *rep *= replication associated protein gene, *cap *= capsid protein gene, Prep = *rep *gene promoter, Ori = origin of replication, core = composite host transcription factor binding site. **B**. Schema of expression cassettes for pTH and pGL3-promoter based plasmid constructs. Whole genome and Pcap PCV-1 DNA inserts were inserted in either orientation into pTHgrttnC, pTHluc and pGL3-promoter plasmids. Plasmids were designated as Cap when whole genome PCV-1 DNA was inserted in the orientation shown in A, or as Rep when the genome was inserted in the opposite orientation. Plasmids were designated as Pcap when Pcap DNA was inserted in the orientation shown in A, or as PcapR when the Pcap fragment was inserted in the opposite orientation. The Pcap core DNA fragment and the CLE fragment (orientation independent hairpin structure) were cloned into pTHgrttnC only. Plasmids were designated as C when the Pcap core fragment was cloned in the orientation shown in A, and as CR when in the opposite orientation.

**Figure 2:**
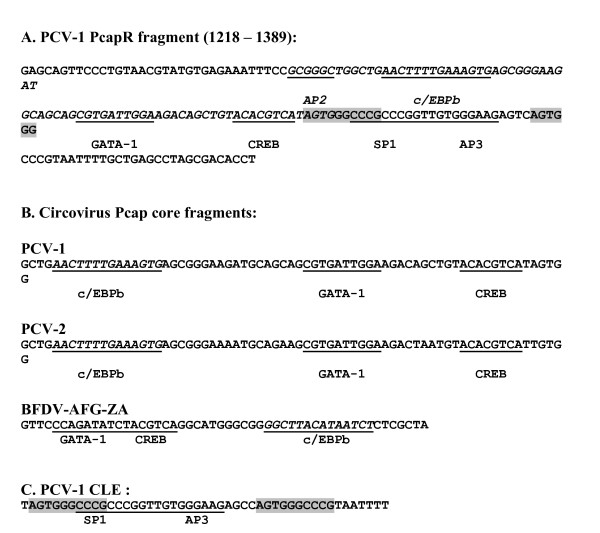
**Sequences of PCV genome and enhancer elements**. **A**. Virion sense DNA sequence of PCV-1, showing the 172 base Pcap fragment in the PcapR (virion sense) orientation. Restriction sites for cloning are not shown. Sequence is numbered as in PCV-1 Accession Number Y09921 [22]. Putative host transcription factor binding sites are indicated and underlined and Conserved Late Element (CLE) motifs are highlighted in grey. The minimal PcapR sequence (1252 - 1328) as identified by Mankertz & Hillenbrand [22] is indicated in italics. **B**. Aligned Pcap core regions from PCV-1, PCV-2 and BFDV-AFG-ZA. PCV-1 sequence includes nt 1260 - 1329. Consensus c/EBPb, GATA-1 and CREB sites are indicated and underlined. **C**. PCV-1 Conserved Late Element (CLE) sequence (47 bp, nt 1324 - 1370): identified according to Cazzonelli *et al. *[32]. Consensus CLE motifs are highlighted in grey. Putative SP1 and AP3 sites are indicated and underlined.

Mammalian DNA viruses with small genomes could be expected to be good sources of regulatory elements for gene expression, and a number of expression control elements derived from mammalian viruses have been tested to date for their expression-enhancing potential in plasmid vaccines [7,8]. The human cytomegalovirus immediate/early promoter/enhancer element (CMV I/E) is the most commonly used promoter (Pcmv) in DNA vaccine plasmids, as it is one of the strongest known promoter elements in mammalian gene expression systems [2,5]. However, there is potential for Pcmv activity to be further improved by the addition of heterologously derived promoter or enhancer sequences. Circoviruses (family *Circoviridae*) are small viruses with circular single-stranded DNA genomes that form histone-bound double-stranded replicative form mini-episomes in the host nucleus and subsequently replicate by rolling circle replication (RCR) [9]. Circoviruses infect avian and porcine hosts and include, among others, Beak and feather disease virus (BFDV) of psittacines [10,11], and Porcine circovirus type 1 (PCV-1) [12,13] and Porcine circovirus type 2 (PCV-2) [14]. PCV-2 is the causative organism for post-weaning multisystemic wasting syndrome (PMWS) in pigs [15,16]. By contrast, PCV-1, originally isolated from the porcine kidney cell line PK-15 [17], is non-pathogenic in any species tested to date including humans [16,18,19]. Like other circoviruses PCV-1 has a compact, genetically dense, bi-directionally transcribed genome of 1759 bp (Figure 1). The ambisense genome of PCV-1 encodes only the genes *cap*, encoding the viral capsid protein, and *rep*, which encodes the replication associated proteins Rep and Rep', the latter being a shorter spliced variant of Rep having the same N-terminus, but with a frame-shifted C-terminus [20,21]. *Rep *and *cap *are divergently transcribed from an 82 bp intergenic region which contains the viral origin of replication (Ori) and the *rep *gene promoter (Prep) which overlaps the viral Ori [12,22]. Prep is negatively regulated by the full length *rep *gene product, Rep [22]. The *cap *promoter (Pcap), shown in Figure 2 is embedded in an intron within the *rep *gene [23], and is activated by host transcription factors [22].

In this study, we investigated the potential utility of the PCV-1 genome as a source of promoter or enhancer sequences for improving antigen expression in an existing HIV-1 subtype C vaccine plasmid, pTHgrttnC, which has been extensively and successfully tested for immunogenicity in both mouse and primate models [3,24,25]. pTHgrttnC encodes a modified synthetic polyantigen, GrttnC, comprising HIV-1C Gag, reverse transcriptase (RT), Tat and Nef sequences fused into a polyprotein. The human codon-optimised *grttnC *sequence is expressed under the control of Pcmv, the CMV intron A and the bovine growth hormone polyadenylation signal on the plasmid vaccine vector, pTH [24,26]. We report here the testing of PCV-1 elements in the test vaccine plasmid pTHgrttnC in terms of their ability to significantly increase GrttnC antigen expression in cell culture and to markedly improve the CTL immune response to an immunodominant GrttnC CD8 epitope in mice, compared to pTHgrttnC. We also report on the expression enhancement of the SV-40 early promoter by a PCV element, and the potential for sequences derived from other circoviruses to enhance expression from the CMV I/E promoter in pTHgrttnC.

## Methods

### Plasmid construction

The PCV-1 genome (identical to GenBank Accession U49186) [27] was obtained by PCR amplification with overlapping primers from cultured porcine kidney cells PK-15 (CCL-33) from the ATCC (KE Palmer, unpublished). A blunt-ended linearised 1759 bp sequence so derived was cloned in pCI (Promega). The PCV-1 genome with *Spe *I sites added on either end was reassembled in pUC18 by polymerase chain reaction (PCR) as detailed previously [28], sequence verified, and sub-cloned into the unique *Spe *I site immediately upstream (5') of and adjacent to the CMV I/E promoter in pTHgrttnC 24. and into the *Nhe *I site immediately upstream of the SV40 early promoter in pGL3-promoter (Promega). Clones were selected containing PCV-1 inserts in either orientation, to yield pTHRepgrttnC and pGL3Rep with the PCV-1 *rep *gene promoter (Prep) in the same sense as the resident promoter, and pTHCapgrttnC and pGL3Cap, with the PCV-1 *cap *gene promoter (Pcap) in the same sense as the resident promoter (see Figure 1).

By combining observations from the Pcap expression study by Mankertz & Hillenbrand [22] with a Transcompel database [29] search for consensus transcription factor (TF) binding sites, we identified a 172 bp DNA fragment containing the PCV-1 capsid promoter region (Pcap). We used PCR to amplify this from the cloned genome and to add *Spe *I sites to both ends, using primers PcapF (5'-TTACTAGTCATATGGAGCAGTTCCCTGTAACG-3') and PcapR (5'-TTACTAGTAGGTGTCGCTAGGCTCAGC-3'). After sequence verification the fragment was sub-cloned in either orientation into *Spe *I-digested pTHgrttnC or *Nhe *I-digested pGL3-promoter, to yield pTHPcapgrttnC and pTHPcapRgrttnC, pGL3Pcap and pGL3PcapR. In pTHPcapgrttnC and pGL3Pcap, Pcap was oriented in the same sense towards the *grttnC *or *luc *transgenes as it would be in PCV-1 for *cap *transcription, as in pTHCapgrttnC or pGL3Cap. In pTHPcapRgrttnC and pGL3PcapR, the Pcap fragment was inserted in the opposite orientation.

We searched the Transcompel database to more narrowly define active transcription-enhancing sequence in Pcap, and identified a 70 bp putative "core" Pcap sequence. This was assembled as a "pre-cut" *Spe *I - flanked heat annealed fragment from an 10 uM equimolar mixture of HPLC-purified phosphorylated oligonucleotides, PCV-1 Fwd-A, PCV-1 Fwd-B, PCV-1 Rev-C, PCV-1/PCV-2 Rev-D (sequences are shown in Table 1), and ligated into *Spe *I-digested pTHgrttnC. Insert presence, number and orientation were determined by PCR, using appropriate combinations of primers PCV-1 Fwd-A and PCV-1/PCV-2 Rev-D, and the pTH-specific primers pTH3F (5'-CGATAGAGGCGACATCAAGC-3') and pTH4R (5'-CCCATAAGGTCATGTACTGG-3'), and sizing PCR products against a 100 bp DNA ladder (Fermentas). Resultant plasmids pTHCgrttnC and pTHCRgrttnC had single copies of the Pcap core, and corresponded in orientation to pTHPcapgrttnC and pTHPcapRgrttnC respectively.

**Table 1:** Oligonucleotides for assembly of Circovirus Pcap cores and PCV-1 CLE

PCV-1 CLE	CLE-Fwd	5'-CTAGCTAGTGGGCCCGCCCGGTTGTGGGAAGAGCCAGTGG GCCCGTAATTTTG - 3'
	CLE-Rev	5'-CTAGCAAAATTACGGGCCCACTGGCTCTTCCCACAACCGG GCGGGCCCACTAG - 3'
PCV-1 Pcap core	PCV-1 Fwd-A	5'-CTAGTCCTAGGCTGAACTTTTGAAAGTGAGCGGGAAGATGCAGCAGCGTG - 3'
	PCV-1 Fwd-B	5'-ATTGGAAGACAGCTGTACACGTCATAGTGGCTAGCA-3'
	PCV-1 Rev-C	5'-CTAGTGCTAGCCACTATGACGTGTACAGCTGTCTTCCAATCACGCTGCTGCATCTTCCCG-3'
	PCV-1/PCV-2 Rev-D	5'-CTCACTTTCAAAAGTTCAGCCTAGGA - 3'

PCV-2 Pcap core	PCV-2 Fwd-P	5'-CTAGTCCTAGGCTGAACTTTTGAAAGTGAGCGGGAAAATGCAGAAGCGTG-3'
	PCV-2 Fwd-Q	5'-ATTGGAAGACTAATGTACACGTCATTGTGGCTAGCA-3'
	PCV-2 Rev-R	5'-CTAGTGCTAGCCACAATGACGTGTACATTAGTCTTCCAATCACGCTTCTGCATTTTCCCG-3'

BFDV Pcap core	BFDV Fwd-W	5'-CTAGTCCTAGGGTTCCCAGATATCTACGTCAGGCATGGGCGGGGCTTAC-3'
	BFDV Fwd-X	5'-ATAATCTCTCGCTAGCTAGCAGCTAGCA-3'
	BFDV Rev-Y	5'-CTAGTGCTAGCTGCTAGCTAGCGAGAGATTATGTAAGCCCCGCCCATGC-3'
	BFDV Rev-Z	5'-CTGACGTAGATATCTGGGAACCCTAGGA-3

Whole-genome sequence alignments (DNAMAN Lynnon Biosoft ver. 6, Quebec, Canada) between PCV-1 (U49186), PCV-2 (Accession number: AY256460) and a South African isolate of Beak and feather disease virus (BFDV-AFG3-ZA, Accession number AY450443) [11] were used to locate the PCV-1 Pcap core sequence equivalents in PCV-2 and BFDV-AFG3-ZA. Deduced Pcap core sequences from PCV-2 and BFDV-AFG3-ZA were assembled as above, using oligonucleotides PCV-2 Fwd-P, PCV-2 Fwd-Q, PCV-2 Rev-R and PCV-1/PCV-2 Rev-D for the PCV-2 core (Table 1) and oligonucleotides BFDV Fwd-W, BFDV Fwd-X, BFDV Rev-Y and BFDV Rev-Z for the BFDV-AFG3-ZA core (Table 1). The assembled sequences were cloned into pTHgrttnC as above, and insert detection was carried out as above, using appropriate combinations of primers pTH3F, pTH4R, PCV-2 Fwd-P, PCV-1/PCV-2 Rev-D, BFDV Fwd-W, or BFDV Rev-Z, to yield the analogous plasmids, pTHC2grttnC and pTHC2RgrttnC (corresponding to Pcap and PcapR orientations, respectively) for PCV-2. pTHgrttnC constructs containing either a monomer or a trimer of the BFDV-AFG3-ZA Pcap core sequence were similarly selected, yielding pTHCBgrttnC and pTHCB3grttnC.

Because of the similarity in genetic organisation and phylogenetic relationship between circoviruses and geminiviruses [30], we used DNAMAN ver. 6 to search the PCV-1 genome for transcription-enhancing DNA sequence repeats, as previously identified for geminiviruses [31]. We identified a 47 bp PCV-1 fragment within our cloned PCV-1 Pcap sequence that contained a consensus "Conserved Late Element" (CLE) sequence [31,32], which we assembled with flanking "pre-cut" *Nhe *I sites from oligonucleotides CLE-Fwd and CLE-Rev (Table 1) and cloned into *Spe *I - cut pTHgrttnC as above. Using primers pTH3F, pTH4R and CLE-Fwd as above, we selected plasmids pTHCLEgrttnC (containing a single copy CLE insert) and pTHCLE3grttnC (containing a CLE trimer).

The *grttnC *ORF was removed from pTHgrttnC by *Hind *III-*Eco *RI digestion and replaced with *Hind *III-*Eco *RI-digested *luc *from pGL3-promoter, to yield pTHluc. The *Spe *I-flanked Pcap fragment was cloned in either orientation into *Nhe *I-digested pTHluc to give pTHPcapluc and pTHPcapRluc.

### HEK293 cell transfection and protein expression

HEK293 cells (CRC-1573) were maintained in 6-well culture plates in Dulbecco's Modified Eagle's Medium (DMEM) with 10% FBS, 100 U/ml penicillin and 100 μg/ml streptomycin. Transfections were performed 24 h post plating at 50% to 70% confluence using 1 ug endotoxin-free DNA (prepared from *E. coli *DH5α cells, using Qiagen Endofree plasmid kit) and either 3 μ FuGENE6 (Roche) transfection reagent or its exact homologue Mirus-LT1 (Mirus). To normalise transfection for those reactions detecting *grttnC *expression 50 ng pcDNA3.1/Zeo/CAT DNA (Invitrogen) which encodes the chloramphenicol acetyl transferase (CAT) gene was included in the reaction. Cells were harvested at 48 h post-transfection and lysed in appropriate buffers for detection of HIV-1 p24 antigen (p24 ELISA, Vironostika, Biomerieux) to detect the GrttnC polyantigen, luciferase (Luciferase Assay System, Promega) and CAT (CAT ELIZA, Roche) respectively, according to kit instructions. p24 levels were normalised against co-expressed CAT levels. Data is reported as the mean responses for 6 replicate transfection reactions.

GrttnC polyprotein in cell lysates expressed from pTHgrttnC-based plasmids was detected by western blot [28] using anti-RT sheep antiserum (ARP428, NISBC Centralised Facility for AIDS reagents, MRC, UK). HIV-1_HXB2 _RT dimer (51 kDa, 66 kDa; NIH AIDS Research & Reference Reagent Programme, McKesson BioServices Corporation, USA) served as a positive control.

### Immunization of mice

We tested the immunogenicity of pTHCapgrttnC, pTHPcapgrttnC, and pTHPcapRgrttnC against pTHgrttnC in 8-10 week old female BALB/c mice. All animal protocols were approved by the Animal Ethics Committee, UCT Faculty of Health Sciences (UCT Animal Research Ethics Committee study reference number 06/012). Initial experiments compared responses to a single 100 μg DNA dose against two 100 μg doses given 28 days apart, with spleens harvested 12 days after the final inoculation. Induction of memory responses by two 10 μg doses of either pTHCapgrttnC, pTHgrttnC or pTH with no insert, given 28 days apart, was evaluated by the ability of SAAVI MVA-C (MVA; 10^4 ^pfu) - a modified vaccinia strain Ankara vector which encodes the GrttnC polyantigen [24] - to boost the response. MVA was given on day 56 and spleens harvested on day 68. Endotoxin-free DNA in PBS (Sigma) for murine immunization was prepared using either the Endofree plasmid preparation kit (Qiagen) following manufacturer's instructions or manufactured commercially (Aldevron, Fargo, USA). MVA was at 10^4 ^pfu per 100 μl in 1 mM Tris, pH 9.0 as detailed previously [3,25].

Groups of female BALB/c mice (5 mice per group) were immunized after anaesthaesia with ketamine:xylazine (10:1). Plasmid (100 μg or 10 μg) and MVA (10^4 ^pfu) inoculations [3,24] were given as intramuscular injections in a final volume of 100 μl with 50 μl injected into each hind leg muscle.

### IFN-γ ELISPOT assay

The number of IFN-γ secreting CD8+ T cells responding to the RT CD8 peptide, as a measure of the response to GrttnC, was evaluated in an IFN-γ ELISPOT assay (Mouse IFN-γ ELISPOT set; BD Pharmingen). Spleens from each group were pooled and a single cell suspension of splenocytes was prepared and treated with erythrocyte lysing buffer (0.15 M NH_4_Cl, 10 mM KHCO_3_, 0.1 mM Na_2_EDTA) for 1 min at room temperature. Splenocytes were plated in triplicate at 500 000 cells/well in a final volume of 200 μl R10 medium (RPMI-1640 with 10% heat inactivated FCS, 15 mM β-mercaptoethanol, 100 U penicillin per ml, and 100 μg streptomycin) to determine the background response or with the H-2K^d^-restricted RT peptide VYYDPSKDLIA (> 95% pure peptide, Bachem, Switzerland) at 4 μg/ml [24]. Spots were detected using Nova Red substrate (Vector Labs) then scanned and counted using a CTL Analyzer (Cellular Technology, OH, USA) with Immunospot Version 3.2 software. The mean number of spots from triplicate wells ± (standard deviation) SD was calculated and background spots (not more than 30 ± 10 sfu/10^6 ^splenocytes) were subtracted and adjusted to spot forming units (sfu) per 10^6 ^splenocytes to give sfu/10^6 ^splenocytes ± SD.

### Statistical analysis

Data was statistically analyzed using Student's *t *test. *P *values of <0.05 were considered significant.

## Results

### Constructs

For ease of making comparisons between studies the PCV-1 DNA sequences used in this study were numbered to match the PCV-1 sequence Y09921 [12]. Thus, the PCV-1 genome cloned into pTHRepgrttnC, pTHCapgrttnC, pGL3Rep and pGL3Cap was linearised within the capsid gene between nucleotides (nt) corresponding to 151 and 152 of Y09921, disrupting the capsid gene (*cap*) such that the sequence coding for the C-terminal 39 aa was separated from the rest of *cap *(Figure 1A). The 172 bp PCV-1 Pcap fragment used in this study corresponded to nt 1389-1218 of Y09921, and is depicted in Figure 2A. The chosen fragment size was longer than the minimal Pcap region (1328 - 1252) mapped by Mankertz & Hillenbrand [22], in order to maximise the inclusion of previously unidentified putative TF binding sites identified using Transcompel in the current study. The PCV-1 Pcap core corresponded to nt 1260 - 1329 of Y09921 (Figure 2B), and the CLE corresponded to nt 1324 - 1370 of Y09921 (Figure 2C). The PCV-2 Pcap core corresponded to nt 451 - 520 of AY256460. The BFDV-AFG3-ZA Pcap core sequence corresponded to nt 390 - 439 of AY450443.

The 70 bp "core sequence" of Pcap includes a putative composite host cell TF binding site, comprising C/EBPb, GATA-1 and CREB recognition/binding sites, as identified from the Transcompel database. Aligned Pcap core sequences from PCV-1, PCV-2 and BFDV-AFG-ZA are shown in Figure 2B. The order of the component putative C/EBPb, GATA-1 and CREB recognition/binding sites differs between the porcine and avian circoviruses. The putative composite TF site arrangement in the BFDV Pcap sequence is typical of that found in avian circoviruses.

### GrttnC antigen expression in HEK293 cells

**Figure 3:**
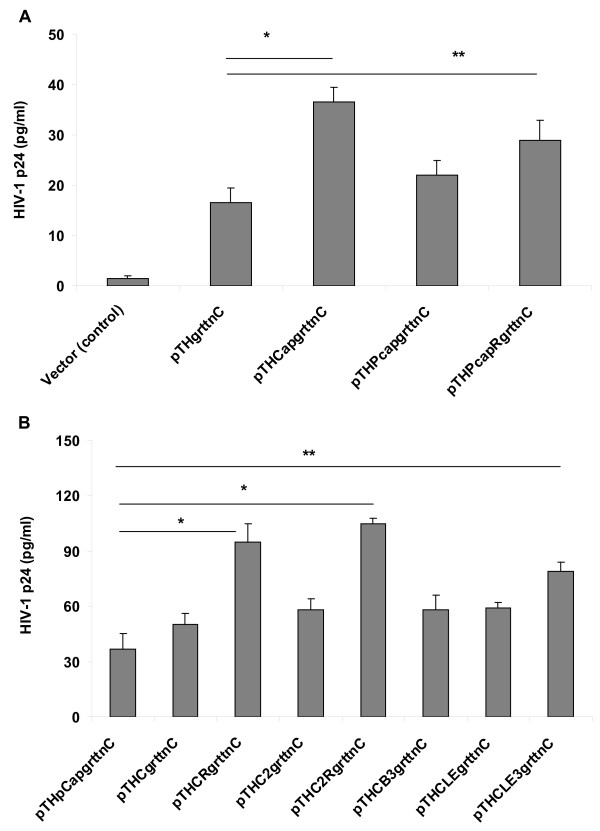
**GrttnC polyantigen expression in HEK293 cells**. HEK293 cells were co-transfected for 48 h with the indicated plasmids and pcDNA3.1Zeo/CAT. HIV-1 p24 antigen was quantified in cell lysates as the measure of GrttnC polyantigen content and normalised using CAT expression. Each data point is the mean of 6 replicated transfections ± SD. **A: **Plasmids expressing GrttnC with and without PCV-1 whole genome or Pcap or PcapR DNA sequence. pTHCap minus insert served as the vector control. **B**: Plasmids expressing GrttnC and containing Pcap elements from either PCV-1 (pTHPcapgrttnC, pTHCgrttnC and pTHCRgrttnC), PCV-2 (pTHC2grttnC and pTHC2RgrttnC) or BFDV-AFG-ZA (pTHCB3grttnC, pTHCLEgrttnC and pTHCLE3grttnC). * p < 0.01; **p < 0.05.

Western blots probed with an RT antiserum indicated pTHCapgrttnC, pTHPCapgrttnC and pTHPCapRgrttnC expressed a protein with an apparent molecular weight identical to that expressed by pTHgrttnC (Additional File 1 Figure S1). Although pTHRepgrttnC showed enhanced GrttnC expression over pTHgrttnC in early experiments, the construct was genetically unstable. For this reason it was not used in further experiments. GrttnC polyantigen expression quantified using the p24 antigen ELISA indicated pTHCapgrttnC - with the whole genome PCV-1 insert - expressed a 2.1 fold higher level of p24 than the parent pTHgrttnC (p < 0.01) (Figure 3A). The 172 bp PCV-1 Pcap fragment did not significantly influence GrttnC expression; however, the PCapR fragment induced a 1.5 fold higher level (p < 0.05) of GrttnC expression than pTHgrttnC (Figure 3A). We next compared GrttnC expression of pTHpCapgrttnC with the following constructs: pTHCgrttnC and pTHCRgrttnC, containing PCV-1 Pcap core sequence and corresponding in orientation to pTHPcapgrttnC and pTHPcapRgrttnC respectively; pTHC2grttnC and pTHC2RgrttnC, containing PCV-2 Pcap core sequence; pTHCB3grttnC, a trimer of the deduced BFDV Pcap core, and pTHCLEgrttnC and pTHCLE3grttnC, containing a CLE monomer or trimer respectively (Figure 3B). Significantly higher expression of GrttnC (p < 0.01) was only achieved with plasmids pTHCRgrttnC (2.4 fold) and pTHC2RgrttnC (2.5 fold) (Figure 3B). GrttnC expression for pTHCLE3grttnC was 1.9 fold greater than pTHpCapgrttnC (p < 0.05) (Figure 3B).

### Luciferase expression in HEK293 cells

In order to test whether the PCV-1 sequence enhanced gene expression in promoter contexts other than the CMV I/E promoter in pTH, we constructed plasmids pGL3Cap, containing whole-genome PCV-1 DNA, and pGL3PCap and pGL3PCapR, containing PCV-1 PCap DNA, where the PCV-1 DNA was inserted immediately upstream of the SV40 early promoter (Figure 1B). Plasmids pTHluc, pTHPcapluc and pTHPcapRluc (Figure 1B) were also constructed to compare luciferase expression by Pcap-CMV I/E and Pcap-SV40 dual promoter constructs. This would also test the ability of the PCV-1 Pcap-CMV I/E promoter combination to express transgenes other than *grttnC*.

**Figure 4:**
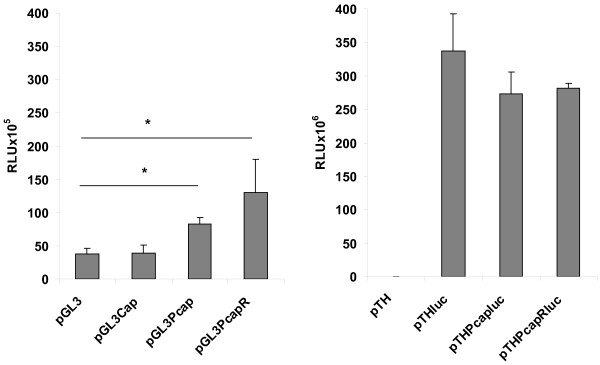
**Luciferase expression in HEK293 cells**. HEK293 cells were transfected for 48 h with the indicated plasmids containing the PCV-1 whole genome or Pcap DNA sequence inserted immediately upstream of either the SV40 promoter or CMV I/E promoter. Luciferase was quantified in cell lysates and each data point is the mean of 6 replicated transfections ± SD. * p < 0.01, **p < 0.05.

Significantly greater luciferase expression above that of the parent plasmid pGL3-promoter (p < 0.01) was achieved only with pGL3Pcap (2 fold) and pGL3PcapR (3 fold) (Figure 4). The CMV I/E promoter-based plasmids also expressed luciferase; however, there was no difference in luciferase levels expressed by pTHluc, pTHPCapluc and pTHPCapR (Figure 4). The CMV I/E promoter-based plasmids expressed a higher average level of luciferase (25 fold; p < 0.01) than pGL3Pcap and pGL3PcapR (Figure 4).

### Immunogenicity

**Figure 5:**
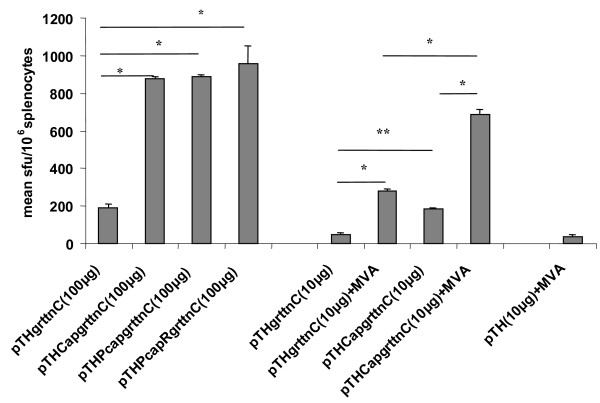
**IFN-γ ELISPOT responses to the RT CD8 peptide**. Groups of BALB/c mice were intramuscularly vaccinated with the indicated DNA vaccine doses on day 0 and day 28. Two groups of mice that were inoculated with 10 μg of DNA were subsequently boosted intramuscularly with MVA (SAAVI MVA-C, 10^4 ^pfu) on day 56. A separate group of mice was vaccinated with pTH (10 μg) minus insert on day 0 and day 28 then boosted with MVA (10^4 ^pfu) on day 56. For all groups spleens were harvested 12 days after the last vaccination and splenocytes used in an IFN-γ ELISPOT assay with the RT CD8 peptide. Responses for mice vaccinated with a DNA dose of 100 μg or 10 μg are the mean sfu/10^6 ^splenocytes ± standard deviation (SD) for n = 5 or n = 3 experiments. * p < 0.01; **p < 0.05.

Responses to the polyantigen grttnC were determined as the response to the RT CD8 peptide in the IFN-γ ELISPOT assay, as this amino acid sequence is known to be an immunodominant epitope [24]. For all plasmids a single 100 μg DNA dose induced low responses of less than 30 net sfu/10^6 ^splencoytes to the RT CD8 peptide (not shown). Vaccination with 2 doses of pTHCapgrttnC, pTHPcapgrttnC or pTHPcapRgrttnC 28 days apart elicited similar RT peptide responses ranging from 879 ± 8 - 889 ± 96 sfu/10^6 ^splenocytes (n = 5) (Figure 5). These responses were approximately 4.6 fold (p < 0.01) superior to the RT peptide response (193 ± 19 sfu/10^6 ^splenocytes; n = 5) induced by 2 vaccinations of pTHgrttnC (Figure 5). The effect of decreasing the dose of pTHCapgrttnC on the response to the RT peptide was investigated (Figure 5). Two 10 μg doses of pTHCapgrttnC given 28 days apart induced a 3.5 fold (p < 0.05) higher level of RT-specific CD8+ T cells than that elicited by two 10 μg pTHgrttnC doses. These low priming doses of pTHCapgrttnC and pTHgrttnC induced memory cells that could expand significantly (p < 0.01) in response to a boost with MVA given on day 56 (Figure 5). The DNA prime MVA boost vaccination regimen induced a response that was significantly greater (p < 0.01) than the sum of the responses to the respective individual DNA and MVA vaccines (Figure 5).

## Discussion

A linearised PCV-1 genome inserted in either orientation immediately upstream of the resident CMV I/E promoter (Pcmv) in the proven DNA vaccine pTHgrttnC significantly improved expression of the HIV-1C-derived polyantigen GrttnC. Early results suggested that genome orientation influenced expression through PCV-1 *rep *(pTHRepgrttnC) or *cap *(pTHCapgrttnC) promoter effects. Plasmid pTHRepgrttnC was unstable, suggesting that whole-genome orientation influences stability. Therefore, given that the effect was the same with either orientation of the PCV genome, we focused on elucidating the capacity of the PCV-1 capsid promoter Pcap to act as an expression enhancing element in conjunction with strong promoters in mammalian expression plasmids. Our chosen PCV-1 Pcap fragment was larger than the previously identified minimal Pcap [22,23] and included more putative TF binding sites than those previously put forward to explain Pcap activity [22]. When the Pcap fragment was cloned in reverse orientation (PcapR) upstream of Pcmv in pTHgrttnC (pTHPcapRgrttnC), GrttnC expression was enhanced to the same level as when the equivalent whole genome PCV-1 construct (pTHCapgrttnC) was used. This result is compatible with the possibility that PcapR may act as an upstream transcriptional enhancer for Prep in the native circular viral genome: circoviruses have highly compact ambisense genomes and some regions encode more than one function. In particular, in PCV-1 one sequence encodes Pcap, part of the *rep *gene intron, and the coding sequence for the full-length Rep protein [23] and lies approximately 1 Kb upstream of Prep - a feasible distance from Prep for PcapR sequence to act as an upstream transcription enhancing sequence for the *rep *gene [33]. We speculate that the PcapR sequence in pTHRepgrttnC could have contributed to expression enhancement in pTHRepgrttnC, although this was not investigated in this study.

We identified two sequences within Pcap that enhanced GrttnC expression from Pcmv: these were the "core" sequence and the CLE (Figure 1 and 2). The active Pcap "core" sequence included closely situated consensus DNA binding sites for the transcription factors c/EBPb, the CCAAT/enhancer-binding protein which controls cell cycle progression [34], GATA-1, a constitutive Zn finger transcription factor [35], and CREB, a cAMP response element-binding protein [36] binding sites without AP2, SP1 and AP3 binding sites (Figure 2B). The nucleotide distances between these sites are consistent with them making up a composite TF binding site, as identified in other genes such as rat CYP2D5, with a composite c/EPB-beta/SP1 site [37], and human gonadotropin alpha-subunit, with a composite CREB/GATA-1 site [38]. This putative C/EBPb/GATA-1/CREB composite TF binding site was not identified in a 76 bp minimal Pcap fragment [22], where Pcap activity was tentatively attributed to putative binding sites for AP2, SP1 and AP3 transcription factors. However, our active Pcap "core" fragment excluded AP2, SP1 and AP3 binding sites (Figure 2B), and Pcap promoter activity was previously seen to drop off almost completely [22], concomitant with the deletion of a fragment that we have currently identified as containing the putative GATA-1 and CREB sites. This strongly suggests that the GATA-1 and CREB binding sites, at least, are crucial to Pcap activity.

We tested constructs carrying other circovirus Pcap-cognate core sequences from PCV-2, a pathogenic strain of PCV, and BFDV-AFG-ZA, an avian circovirus. The PCV-1 and PCV-2 Pcap core fragments share 80% sequence identity overall and identical consensus c/EBPb/GATA-1/CREB binding site sequence and spacing. The c/EBPb, GATA-1 and CREB TF consensus sequence and site arrangement in the BFDV-AFG-ZA Pcap core is typical of avian circoviruses but different from that of PCV-1 and PCV-2. Equally improved expression occurred with pTHCRgrttnC (PCV-1) and pTHCR2grttnC (PCV-2). It thus appears the order and spacing of TF binding sites in the composite TF binding site, but not the specific nucleotide sequence between sites, was important to overall Pcap core activity in mammalian cells, at least in the current *in vitro *expression system.

Lying adjacent to the "core" sequence in our chosen Pcap fragment was a direct repeat of a consensus sequence AGTGGGCCCG separated by 19 nt and capable of forming a hairpin structure. This was identical to the transcription enhancing Conserved Late Element (CLE), first identified as an element in the geminiviruses, which are distantly evolutionarily related to circoviruses, but which infect plants [39]. The CLE was later found to be a native upstream transcription-enhancing element in a number of bacterial and plant genes, showing additive increase in activity with number of CLE units [31,32]. In a sequence database search performed in the current study we made the novel observation that CLE consensus sequences may be found in the DNA binding sites of zinc finger containing proteins in a number of genes from all phyla.

We showed that a concatenated PCV-1 CLE trimer enhanced GrttnC expression over that of pTHPcapgrttnC, the first demonstration that an identified CLE enhances gene expression in a mammalian expression system. It has been observed elsewhere [22] that maximal Pcap expression activity was associated with a sequence that included putative SP1 and AP3 binding sites (to which the maximal activity was attributed), but which coincidentally included the complete CLE. The 47 bp CLE - containing fragment tested and found active in the present study encompasses the putative SP1 and AP3 binding sites previously indicated [22], and so a contribution from these TFs cannot be ruled out.

To exclude the possibility that the observed expression enhancement in plasmids containing Pcap or its derivatives was fortuitous, we tested three other PCV-1 sub-genomic DNA fragments in pTHgrttnC and pGL3-promoter for transgene expression enhancement. These fragments, ranging in size from 266 - 316 bp, included putative TF binding sites as identified via Transcompel, other than any previously identified *rep *or *cap *promoter sequences. None of these fragments in either orientation showed any transgene expression enhancing ability in pTHgrttnC or pGL3-promoter (not shown).

On testing PCV-1 promoters in a different transgene or promoter context, we observed that PCV-1 promoters in whole genome inserts in pGL3-promoter appeared to be too distant from the SV40 promoter to affect luciferase expression, but the isolated Pcap fragment brought Pcap into closer proximity with the SV40 promoter, permitting enhanced luciferase expression. The stronger CMV I/E promoter [5,8] gave approximately 25 fold more expression in the pTH context than the SV40 promoter in the pGL3-promoter context. Luciferase expression in pTHluc was not increased by a Pcap or PcapR insert. Our experience with other expression systems [28,40] is that one gene insert may be expressed at significantly higher levels than another from the same gene control elements. Thus, the specific contribution of the PCV-1 Pcap element should be empirically determined for each new transgene tested.

The PCV-1-containing vaccine plasmids pTHCapgrttnC, pTHPcapgrttnC and pTHPcapRgrttnC showed significantly improved immunogenicity compared with the parent plasmid, pTHgrttnC. Use of pTHCapgrttnC clearly showed the potential for dose sparing, in that 10 ug doses elicited CTL responses in mice that were comparable to 100 ug doses of pTHgrttnC. This effect was further improved with an MVA-grttnC boost. Thus, even at low doses, these DNA vaccines elicited memory cells capable of responding to a matched booster vaccine.

A potential safety concern with using full length PCV-1 genome-containing plasmids as DNA vaccines is that the Rep protein encoded in the PCV-1 genome might bind to essential host factors involved in regulation of basic cellular functions. For example, Rep of the related PCV strain, PCV-2, was shown *in vitro *to bind *c-myc*, a multi-functional transcription factor involved with aspects of cell regulation, including cell proliferation and apoptosis [41]. This concern could potentially apply to pTHCapgrttnC. Thus it is encouraging that pTHPcapgrttnC and pTHPcapRgrttnC showed similar immunogenic potential to pTHCapgrttnC. The 172 bp PCV-1 Pcap sequence included in these constructs has no involvement with *c-myc *binding, and so these plasmids are free of the above potential safety concern.

## Conclusions

In summary, we have shown the potential for the PCV-1 capsid gene promoter and other circovirus-derived elements to act as an expression enhancing elements in conjunction with strong mammalian promoters, by forming a dual promoter with either the CMV I/E promoter or the SV40 early promoter. We traced Pcap activity to a 70 bp Pcap "core" fragment containing a putative composite binding site for the host cell transcription factors c/EBPb, GATA-1 and CREB, and to a 47 bp fragment containing a CLE sequence that lies adjacent to the "core" in the native Pcap of PCV-1. We showed that a Pcap core sequence from the related circovirus PCV-2 acts in the same way as that from PCV-1 while an avian circovirus "core" sequence showed little activity in a mammalian cell line, possibly as a result of the different order and sequence of the consensus c/EBPb, GATA-1 and CREB binding sites.

The major potential advantage of using a DNA vaccine construct containing PCV sequences lies in dose-sparing - an advantage in human trials where conventionally up to 5 mg of plasmid vaccine is used. Alternatively, higher immune responses could be achieved with the same doses of the enhanced DNA vaccine compared to already effective conventional versions: recent evidence that higher doses of DNA vaccines given with genetic adjuvants elicit potent CTL responses mean that our PCV-derived enhancer may be even more effective than in our present demonstration [42-44].

## Abbreviations Used

BFDV: Beak and feather disease circovirus; CMV: cytomegalovirus; CTL: cytotoxic T-lymphocyte; ELISPOT: enzyme-linked immunosorbent spot (assay); Grttn: HIV-1 Gag-RT-Tat-Nef polyprotein; HIV-1: Human immunodeficiency virus type 1; I/E: immediate early (promoter); Ifnγ: interferon gamma; PCR: polymerase chain reaction; PCV-1: Porcine circovirus type 1; Pcap, Prep: capsid and replication-associated protein promoters; TF: transcription factor.

## Competing interests

The authors declare that they have no competing interests, other than a pending patent application on the use of circovirus and other ssDNA virus enhancer elements (FT and EPR).

## Authors' contributions

FT and A-LW and EPR conceived the study; FT planned and performed all molecular biological work, with assistance from MB, and helped ES plan the immunological studies. ES planned and supervised all mouse work and immunological procedures and assays. KEP provided the PCV-1 genomic clone and preliminary expression results, and assisted in editing the paper. The paper was written and edited by FT and ES and EPR. All authors read and approved the final manuscript.

## Supplementary Material

Additional file 1 **Confirmation of GrttnC expression by pTHGrttnC derived plasmids carrying PCV-1 DNA inserts**. Extracts from HEK293 cells transfected with pTHgrttnC-based plasmids (loading volumes not adjusted for transfection efficiency) were separated on 10% polyacrylamide, electroblotted onto nitrocellulose and probed for the RT component of GrttnC with RT specific antiserum (ARP 428). Lane 1. Precision Plus Kaleidoscope MWt marker. Lane 2. Positive control protein = 200 ng HIV-1_HXB2 _Reverse Transcriptase dimer (51 kDa, 66 kDa). Lane 3. pTHCap (empty vector). Lane 4. pTHgrttnC. Lane 5. pTHCapgrttnC. Lane 6. pTHPcapgrttnC. Lane 7. pTHPcapRgrttnC. Click here for file
