# Supplementary figures and images for: The porcine circovirus type 1 capsid gene promoter improves antigen expression and immunogenicity in a HIV-1 plasmid vaccine
Source: Virol J. 2011 Feb 7;8:51. doi: 10.1186/1743-422X-8-51 (PMC3041773; doi:10.1186/1743-422X-8-51)

## Slide 1
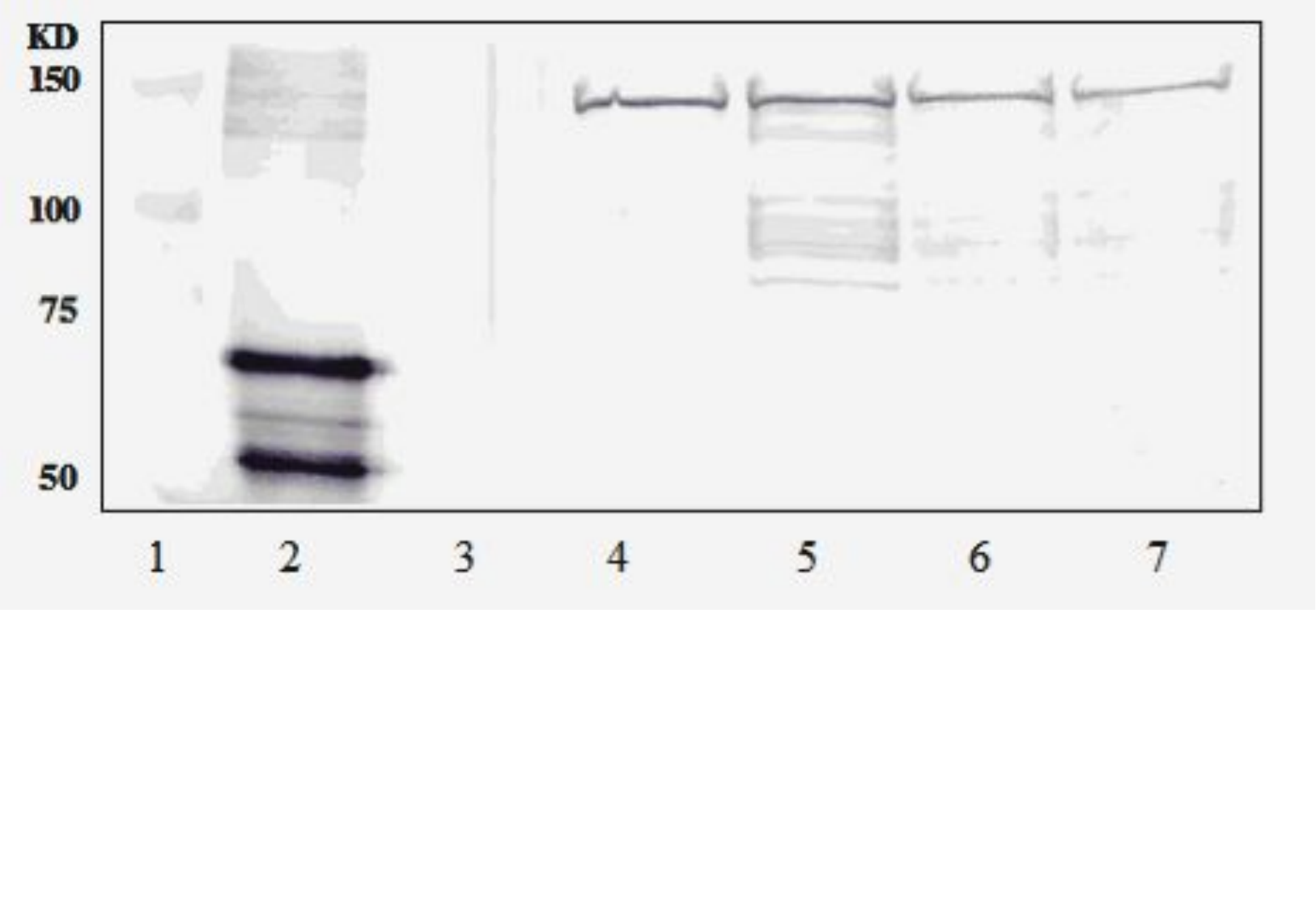

Supplement: Additional file 1 — Confirmation of GrttnC expression by pTHGrttnC derived plasmids carrying PCV-1 DNA inserts. Extracts from HEK293 cells transfected with pTHgrttnC-based plasmids (loading volumes not adjusted for transfection efficiency) were separated on 10% polyacrylamide, electroblotted onto nitrocellulose and probed for the RT component of GrttnC with RT specific antiserum (ARP 428). Lane 1. Precision Plus Kaleidoscope MWt marker. Lane 2. Positive control protein = 200 ng HIV-1HXB2 Reverse Transcriptase dimer (51 kDa, 66 kDa). Lane 3. pTHCap (empty vector). Lane 4. pTHgrttnC. Lane 5. pTHCapgrttnC. Lane 6. pTHPcapgrttnC. Lane 7. pTHPcapRgrttnC. [file 1743-422X-8-51-S1.PPT]
